# Genetic polymorphisms of STAT3 correlated with prognosis in diffuse large B-cell lymphoma patients treated with rituximab

**DOI:** 10.1186/1475-2867-14-25

**Published:** 2014-03-13

**Authors:** Yunfei Hu, Ning Ding, Xuan Jin, Lixia Feng, Lingyan Ping, Yuqin Song, Jun Zhu

**Affiliations:** 1 Key laboratory of Carcinogenesis and Translational Research (Ministry of Education), Department of Lymphoma, Peking University Cancer Hospital & Institute, No.52 Fucheng Road, Haidian District, Beijing 100142, China; 2 Department of Oncology, Affiliated Hospital of Guiyang Medical College, Guizhou Cancer Hospital, No.1 Beijing West Road, Yunyan District, Guiyang 550003, China; 3 Department of Internal Medicine Oncology, Peking University First Hospital, No.8 Xishiku Street, Xicheng District, Beijing 100034, China

**Keywords:** Diffuse large B-cell lymphoma, STAT3, Single nucleotide polymorphism, Rituximab

## Abstract

**Background:**

Rituximab in the combination of CHOP chemotherapy has been widely used as the standard treatment for several kinds of B-cell non-Hodgkin lymphoma (B-NHL). Inactivation of phosphorylation of *STAT3* plays an essential role in rituximab-induced anti-proliferative activity in B-cell lymphoma. However, the relationship between *STAT3* genetic polymorphisms and clinical response to standard frontline treatment with rituximab has not been well illustrated yet.

**Methods:**

In this study we analyzed the *STAT3* polymorphisms and prognosis of 166 diffuse large B-cell lymphoma (DLBCL) patients who were treated with rituximab from 2007 to 2010. Determination of the *STAT3* polymorphisms of rs2293152 from genomic DNA was achieved by Sanger chain termination sequencing.

**Results:**

We did not observe obvious correlation between patients’ disease features and *STAT3* polymorphisms, but patients with homozygous genotypes at rs2293162 showed a trend of higher CR rate than those with the heterozygous genotype, especially in non-GCB subgroup (*p* = 0.011). Furthermore, homozygous genotypes GG and CC also showed advantages of long-term survival compared with heterozygous genotype patients (*p* = 0.022).

**Conclusions:**

These results suggest that *STAT3* polymorphisms could be a suitable biomarker related to clinical outcome of DLBCL patients treated with rituximab.

## Background

Diffuse large B-cell lymphoma (DLBCL) is the most common B-cell non-Hodgkin lymphoma (B-NHL) subtype [1]. The introduction of rituximab plus cyclophosphamide/doxorubicin/vincristine/prednisone (R-CHOP) chemotherapy is considered as the standard treatment for DLBCL patients, which dramatically improves the treatment outcome and prognosis [2]. However, a great number of B-NHL patients treated with this immunotherapy still develop primary and secondary resistance to rituximab [3]. Thus, novel prognostic factors are being explored to predict treatment outcome.

*STAT3* is a member of the signal transducer and activator of transcription (STAT) family, which regulates many cellular and biological processes such as cell proliferation, division, and apoptosis via mediation of related genes expression [4,5]. Constitutively activated *STAT3* has been demonstrated to be essential for the proliferation and survival of DLBCL cells, which provides a prognostic indicator and ideal target for DLBCL treatment [6,7]. Despite the activation of antibody-dependent cellular cytotoxicity (ADCC) and complement-dependent cytotoxicity (CDC), many studies have demonstrated that rituximab could also induce apoptosis by inhibiting phosphorylation of *STAT3* in DLBCL cells [8,9]. We hypothesized that polymorphic differences in *STAT3* may account for distinct clinical efficacy of rituximab in DLBCL patients.

In our study, initially several important single nucleotide polymorphisms (SNPs) in *STAT3* (including rs2293152, rs6503695, and rs12949918), which have been associated with gene expression and lymphoma risk [10,11], were analyzed in 166 peripheral blood specimens from DLBCL patients treated with rituximab. During the following investigation of relationships between the polymorphisms and clinical outcome, we eventually identified the prognostic significance of *STAT3* polymorphisms in rs2293152, which could be a suitable predictor related to rituximab efficacy.

## Results

### Patients’ characteristics

The general characteristics of the patients (82 male and 84 female patients) in this study are summarized in Table 1. Sixty-three patients (38.0%) exhibited B symptoms. Eighty-eight patients (53.0%) were in stages 3 or 4, and 50 patients (30.1%) had intermediate-to-high or high international prognostic index (IPI) scores. Bone marrow was involved by lymphoma in 5 patients (3.0%) at diagnosis. R-CHOP or R-CHOP like treatment as a frontline regimen was administrated to 166 patients whose clinical efficacy was evaluable for this study.

**Table 1:** Patient’s characteristics and their correlations with STAT3 polymorphism genotypes

**Clinical parameters**	**No.**	**Genotype**		***p***	**Clinical parameters**	**No.**	**Genotype**		***p***
		**CC + GG**	**CG**				**CC + GG**	**CG**	
**Gender**					**IPI score**				
**Male**	82	50	32	0.042	**0-2**	116	61	55	0.867
**Female**	84	38	46		**3-5**	50	27	23	
**Age**					**Bulky mass**				
**≤60**	106	59	47	0.364	**≥10 cm**	33	19	14	0.557
**>60**	60	29	31		**<10 cm**	133	69	64	
**B symptoms**					**Localized**				
**Positive**	63	31	32	0.442	**Yes**	27	17	10	0.258
**Negative**	103	57	46		**No**	139	71	68	
**LDH**					**Extra Nodal Site**				
**Positive**	78	39	39	0.464	**≤1**	123	64	59	0.669
**Negative**	88	49	39		**>1**	43	24	19	
**β-2 MG**					**Incidence Site**				
**Positive**	51	23	28	0.091	**Lymph node**	95	46	49	0.170
**Negative**	106	63	43		**Extra lymph**	71	42	29	
**ESR**					**Ki-67**				
**Positive**	77	36	41	0.132	**≤75**	66	32	34	0.329
**Negative**	80	47	33		**>75**	85	48	37	
**Stage**					**Subtype**				
**I-II**	78	42	36	0.839	**GCB**	29	18	11	0.229
**III-IV**	88	46	42		**Non-GCB**	113	56	57	

### *STAT3* polymorphisms

*STAT3* polymorphisms at rs2293152 in 166 patients were identified (Table 2). Thirty-one patients (18.7%) had homozygous CC genotype; 57 patients (34.3%), GG; and 78 patients (47.0%), heterozygous CG. The frequency of the C allele in DLBCL patients was 44.7%, and that of the G allele was 55.3%. The genotype distribution of the DLBCL population enrolled in our study was in Hardy-Weinberg equilibrium with regard to the *STAT3* polymorphisms examined.

**Table 2:** Genotype and allele frequencies of STAT3 polymorphisms in 166 Chinese patients with DLBCL

**SNP**	**Genotype frequencies**									
	**Genotype**	**Freq**	**Count**	**Genotype**	**Freq**	**Count**	**Genotype**	**Freq**	**Count**	**Total**
**rs2293152**	CC	0.187	31	CG	0.470	78	GG	0.343	57	166
**SNP**	**Allele frequencies**									
	Allele	Freq	Count	Allele	Freq		Count		Total	
**rs2293152**	C	0.447	109	G	0.553		135		244	

### Correlation analysis between *STAT3* polymorphisms and clinical features of DLBCL patients

Analysis of *STAT3* polymorphisms of rs2293152 suggested that patients with homozygous CC and GG genotypes had a higher tendency to be male than those with the heterozygous genotype (*p* = 0.042). Furthermore, our data showed that an elevated β2-MG level might be less frequent in patients with homozygous genotypes than in those with the heterozygous CG genotype (*p* = 0.091). The distribution of this *STAT3* polymorphisms did not correlate with the germinal center B cell-like (GCB) and non-GCB subtypes (*p* = 0.229; Table 1).

### Impact of the *STAT3* polymorphisms on the curative effect of rituximab

Of the 166 patients evaluable for response to R-CHOP and R-CHOP-like chemotherapy, the overall response rate (ORR) was 86.1% (143 of 166 patients), including a complete response (CR) rate of 52.4% (87 of 166 patients) and a partial response (PR) rate of 33.7% (56 of 166 patients). As shown in Table 3, higher CR rate was observed in patients with homozygous genotypes than in those with the heterozygous CG genotype (*p* = 0.032). In subgroup analysis based on the GCB/non-GCB molecular types, patients with homozygous genotypes also showed better CR rate than heterozygous genotype in non-GCB DLBCL patients (*p* = 0.011).

**Table 3:** Clinical response to rituximab according to STAT3 polymorphism genotypes

**Response**	**Genotype**		***p***
	**CC + GG**	**CG**	
**All patients**			
**CR**	53(60.2)	34(43.6)	0.032
**PR + PD + SD**	35(39.8)	44(56.4)	
**OR**	77(87.5)	66(84.6)	0.591
**PD + SD**	11(12.5)	12(15.4)	
**GCB subtype**			
**CR**	14(77.8)	8(72.7)	0.758
**PR + PD + SD**	4(22.2)	3(27.3)	
**OR**	17(94.4)	11(100)	0.426
**PD + SD**	1(5.6)	0(0)	
**Non-GCB subtype**			
**CR**	32(57.1)	19(33.3)	0.011
**PR + PD + SD**	24(42.9)	38(66.7)	
**OR**	46(82.1)	46(80.7)	0.844
**PD + SD**	10(17.9)	11(19.3)	

### Relationship between the *STAT3* genotypes and time to progression-free survival and overall survival

**Figure 1:**
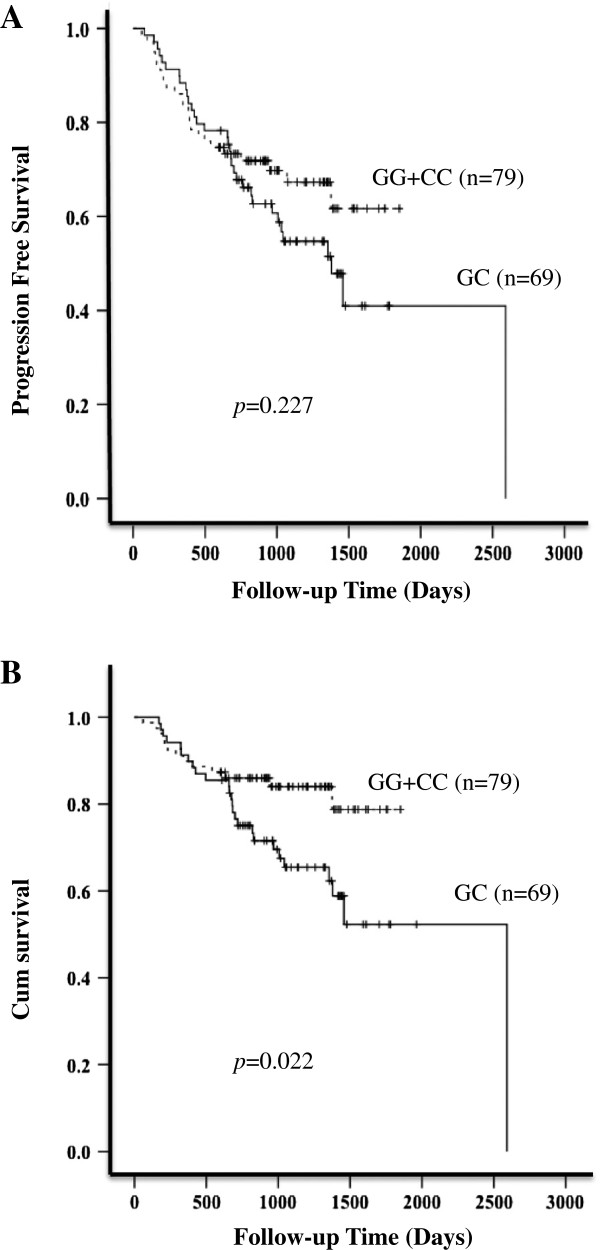
Kaplan-Meier Curves for Progression-free Survival and Overall Survival in DLBCL patients.

After a median follow-up time of 913 days (range, 60–2591 days), 19 (11.4%) patients relapsed or progressed, and 39 (23.5%) died. Six patients were enrolled in a RAD001 maintenance therapy clinical study, and follow-up data were not available for 12 patients. Therefore, a total of 148 patients were evaluated for PFS and OS. Patients with homozygous genotypes had a median PFS of 909 days (range, 60–1851) versus 920 days (range, 78–2591) for the heterozygous CG genotype patients, but the differences between these two groups did not reach statistical significance (*p* = 0.227). Interestingly, a longer OR was observed in patients with homozygous genotypes (range, 60–1851) compared with heterozygous CG genotype patients (range, 170–2591; *p* = 0.022), suggesting that *STAT3* polymorphisms could be useful markers to predict the long-term outcome of rituximab treatment (Figure 1). However, this difference was not observed when patients were subdivided into GCB and non-GCB groups (data not shown).

In the multivariate analysis, we attempted to evaluate the following variables on OS: stage, age, LDH level, β2-microglobulin level, extranodal involvement, bulky mass, IPI score, B symptoms, and *STAT3* polymorphism. This analysis also demonstrated that *STAT3* polymorphism was an important determinant of poor prognosis (*p* = 0.006; HR 2.787; 95% CI 1.346-5.773; Table 4).

**Table 4:** Multivariate analysis of STAT3 polymorphisms on survival

**Variable**	**Hazard ratio**	**95% CI**	***p***
**IPI score**	2.792	1.407 - 5.544	0.003
**STAT3 polymorphisms**	2.787	1.346 - 5.773	0.006
**B symptoms**	0.420	0.210 - 0.839	0.014

## Discussion

In this retrospective analysis, *STAT3* polymorphisms at rs2293152 were found to associate with β2-MG levels and patient sex. Patients with a CC and GG genotypes at rs2293152 could benefit from rituximab-combined chemotherapy.

Rituximab is a single human mouse chimeric IgG antibody, which combines with the CD20 antigen on the surface of DLBCL cells, directly killing these tumor cells [12]. The antitumor activities of rituximab are mainly believed to be associated with the induction of ADCC and CDC [13]. Polymorphisms in *FcγRIIIa*, which plays an essential function in ADCC-induced lymphoma cell lysis, were found to relate to the clinical response of rituximab [14]. In a previous study, our group also demonstrated that polymorphisms in the key CDC regulator, gene *C1qA*, were related to the efficacy of rituximab in the treatment of DLBCL patients [15]. Furthermore, rituximab has also been suggested to induce apoptosis of lymphoma cells by the inhibition of *STAT3* phosphorylation. Various types of tumor cells exhibited constitutively phosphorylated *STAT3* within the nucleus, which has been shown to promote uncontrolled malignant tumor growth and relate with a poor prognosis of patients [16,17]. However, the effect of *STAT3* polymorphisms on the clinical antitumor activities of rituximab has not been reported before. In our study, DLBCL patients with homozygous genotypes in *STAT3* rs2293152 showed better CR rates. Furthermore, the impact of *STAT3* polymorphisms on the treatment efficacy was also analyzed in GCB/non-GCB subgroups. Our results showed that patients with homozygous genotypes also showed higher CR rate than heterozygous genotype in non-GCB DLBCL but not in GCB DLBCL subgroup, which was consistent with previous reports that the activation of *STAT3* plays a more important role in ABC-DLBCL than GCB-DLBCL [18,19]. On the other hand, the *STAT3* polymorphism positions such as rs6503695 and rs12949918 did not show any such relationship with CR and OR rates after rituximab treatment (data now shown).

Previously *STAT3* polymorphisms had been shown to be suitable as predictive makers to analyze the response to IFN-α in renal cell carcinoma [20]. In that study, the SNP site within *STAT3* resulted in a higher expression of *STAT3*, which might contribute to resistance after IFN-α treatment. The *STAT3* SNP site rs2293152 C to G change is an intronic polymorphism, which does not directly introduce amino acid substitution. However, it is increasingly being reported that intron SNPs and silent polymorphisms could also alter the function of target proteins [21,22]. Griseri et al. revealed that a synonymous polymorphism within *TTP* was associated with protein translation and clinical response rate to herceptin treatment in breast cancer patients [23]. An *ERCC1* polymorphism, which induced a codon change from common usage codon AAC to AAT, has also been found to suppress *ERCC1* expression, and the objective response rate was much higher after oxaliplatin and 5-fluorouracil combination chemotherapy in colorectal cancer patients [24]. In the previous analysis of rs2293152 locus of *STAT3*, Sato et al. reported that the CC genotype was found to be associated with the development of Clone Disease. The polymorphisms of *STAT3* in rs2293152 may alter the function of *STAT3*, further activating the inflammatory signaling pathway and occurrence of the disease [25]. Thus, although rs2293152 is a synonymous polymorphism, this single nucleotide change might affect the phosphorylation level of *STAT3*, which could contribute to influence the curative effect of rituximab. However, further experimentation is needed to explore the precise mechanism by which *STAT3* polymorphisms affect the lymphoma responses to R-CHOP chemotherapy.

## Conclusions

The *STAT3* polymorphism could be a predictive biomarker related to clinical outcome of DLBCL patients treated with rituximab.

## Methods

### Patient characteristics

A total of 166 consenting patients who received R-CHOP or R-CHOP-like chemotherapy (R-COP, R-COPP and R-CHOPE) as a frontline regimen between June 2007 and December 2010 were included in this retrospective study from Peking University Cancer Hospital. All patients had CD20^+^ DLBCL according to the World Health Organization classification, as confirmed by our Department of Pathology. Peripheral blood specimens from all lymphoma patients were obtained before the initiation of therapy. R-CHOP chemotherapy was administered as follows: one course of chemotherapy consisted of an intravenous infusion of 750 mg/m^2^ cyclophosphamide, 50 mg/m^2^ adriamycin, and 2 mg vincristine, and oral administration of 100 mg prednisone on days 1 to 5, which was repeated every 3 weeks. Rituximab (375 mg/m^2^) was infused for over 4–6 hours on day 1 before CHOP or CHOP-like chemotherapy was started. The response to R-CHOP therapy was evaluated after completion of 2–3 courses of therapy, 1–2 months after completion of all planned therapy, and then every 3 months for the first year and every 6 months thereafter until progression. This retrospective research protocol was approved by our Institutional Review Board.

### DNA extraction and genotyping

Genomic DNA was isolated from whole blood with the Whole Blood Genome DNA Isolation Kit (Omega Bio-Tek, Doraville, GA, USA) according to the manufacturer’s instructions. DNA was diluted in water to a final stock concentration of 30 ng/μl, and 1 μl was used in each PCR reaction. Determination of *STAT3* polymorphism genotypes was achieved in a blinded manner on coded specimens by Sanger chain termination sequencing. Briefly, the genomic DNA region of interest was amplified using rs2293152 forward primer 5′GGTCACCTACATAGTTGATTG3′ and reverse primer 5′ACACCCCAGTTGTCTTTCATC3′. An initial denaturation step at 94°C for 3 mins was followed by 35 cycles of denaturation at 94°C for 30 s, annealing at 56°C for 30 s, and extension at 72°C for 45 s. The final extension step was for 10 mins. The PCR products were visualized on a 2% agarose gel. All fragments were purified with the AxyPrep DNA Gel Extraction kit (Axygen Sci. Inc., CA, USA). The purified products were sequenced using an ABI 3730XL Avant Genetic Analyzer (Applied Biosystems Inc., CA, USA).

### Definitions

Clinical responses were determined by physical examination and confirmed by computed tomography or ultrasonography. The latter was only used for evaluating superficial lymph nodes. The responses were scored according to the International Working Group criteria. Overall survival (OS) was measured from day 1 of the first cycle of R-CHOP until death from any cause or the last follow-up available. The progression-free survival (PFS) was calculated from day 1 of the first cycle of R-CHOP to disease progression or death from any cause.

### Statistical analysis

The clinical characteristics and response rate of the patients were compared using chi-square and Fisher’s exact tests. The Kaplan-Meier method was used to estimate the differences of PFS and OS. The Cox regression model was used to evaluate the prognostic factors. Differences between groups were regarded as significant at *p* < 0.05. SPSS16.0 was used for all statistical analysis.

## Competing interests

The authors declare that they have no competing interests.

## Authors’ contributions

JZ and YS designed the study and review the final manuscript. YH and ND performed experiment and prepared manuscript. XJ, LF and LP helped to collect specimens and clinical information. YH and ND contribute equally to this work. All authors read and approved the final manuscript.
